# The Morbid Effects of the Retention in the Blood of the Elements of the Urinary Secretion

**Published:** 1861-10

**Authors:** 


THE MORBID EFFECTS OF THE RETENTION IN THE BLOOD OF THE ELEMENTS OF THE URINARY SECRETION. By WILLIAM WALLACE MORELAND, M. D., Member of the Boston Society for Medical Improvement; one of the Attending Surgeons at the Central Office of the Boston Dispensary, etc. Being the Dissertation to which the Fiske Fund Prize was Awarded, July 11, 1860. Philadelphia: Blanchard and Lea. 1861.

This Essay, originally printed in the *American Journal of the Medical Sciences*, is another of the very excellent and valuable contributions made to American medical literature, through the agency of the Fiske Prize Fund of the Rhode Island Medical Society. No branch of pathological investigation has been more assidously or enthusiastically cultivated than this which Prof. Hammond styles *Urology*,—nor is there any which has better repaid the researches of the student, or thrown more light upon an all-pervading and powerful source of disease. What the stethoscope and the speculum have done for other organs, the test-tube and the microscope have accomplished for those equally important ones,—the kidneys, the great blood-depurators of the animal economy. They have furnished us a key to the many mysteries which envelope those diseases, resulting from a retention of the elements of the urine in the blood. They have extorted from the secretion the secret of its composition, and have enabled the physician to hold to strictest account these emunctories; so that their failure to deliver up in due proportion any one of its constituents, made manifest, rationally, by disturbance in some other organ, may be proved, physically, and with mathematical precision, by an appeal to the *experimentum crucis* of the acid and the lens.

Dr. MORLAND has here given us a condensed résumé of our present information on the subject; and, to the great convenience of those desireous of still more detailed and thorough knowledge, a most ample wealth of reference and quotation. The work is one, not admitting of further condensation or abridgement, from its already cyclopœdic brevity and character; we therefore, content ourselves with this notice of it, and recommend it to our readers for perusal. W. B. Keen, 148 Lake Street, Chicago.

